# The changes in renal function after a single dose of intravenous furosemide in patients with compensated liver cirrhosis

**DOI:** 10.1186/1471-230X-6-39

**Published:** 2006-11-29

**Authors:** Nimer Assy, Mohib kayal, Yoram Mejirisky, Miguel Gorenberg, Osamah Hussein, Sorina Schlesinger

**Affiliations:** 1 Liver Unit, Sieff Hospital, Safed, Israel; 2 Department of Internal Medicine A, Sieff Hospital, Safed, Israel; 3 Department of Internal Medicine B, Sieff Hospital, Safed, Israel; 4 Department of Nuclear Medicine, Sieff Hospital, Safed, Israel; 5 Faculty of Medicine, Technion Institute of Technology, Haifa, Israel

## Abstract

**Background:**

Patients with compensated Child-A cirrhosis have sub clinical hypovolemia and diuretic treatment could result in renal impairment.

**Aim:**

To evaluate the changes in renal functional mass as reflected by DMSA uptake after single injection of intravenous furosemide in patients with compensated liver cirrhosis.

**Methods:**

Eighteen cirrhotic patients were divided in two groups; eight patients (group 1, age 56 ± 9.6 yrs, Gender 5M/3F, 3 alcoholic and 5 non alcoholic) were given low intravenous 40 mg furosemide and ten other patients (group 2, age 54 ± 9.9, Gender 6M/4F, 4 alcoholic and 6 non alcoholic) were given high 120 mg furosemide respectively. Renoscintigraphy with 100MBq Of Tc 99 DMSA was given intravenously before and 90 minutes after furosemide administration and SPECT imaging was determined 3 hours later. All patients were kept under low sodium diet (80mEq/d) and all diuretics were withdrawn for 3 days. 8-hours UNa exertion, Calculated and measured Creatinine clearance (CCT) were performed for all patients.

**Results:**

Intravenous furosemide increased the mean renal DMSA uptake in 55% of patients with compensated cirrhosis and these changes persist up to three hours after injection. This increase was at the same extent in either low or high doses of furosemide. (From 12.8% ± 3.8 to 15.2% ± 2.2, p < 0.001 in Gr I as compared to 10.6% ± 4.6 to 13.5% ± 3.6 in Gr 2, p < 0.001). In 8 patients (45%, 3 pts from Gr 1 and 5 pts from Gr 2) DMSA uptake remain unchanged. The mean 8 hrs UNa excretion after intravenous furosemide was above 80 meq/l and was higher in Gr 2 as compared to Gr 1 respectively (136 ± 37 meq/l) VS 100 ± 36.6 meq/l, P = 0.05). Finally, basal global renal DMSA uptake was decreased in 80% of patients; 22.5 ± 7.5% (NL > 40%), as compared to normal calculated creatinine clearance (CCT 101 ± 26), and measured CCT of 87 ± 30 cc/min (P < 0.001).

**Conclusion:**

A single furosemide injection increases renal functional mass as reflected by DMSA in 55% of patients with compensated cirrhosis and identify 45% of patients with reduced uptake and who could develop renal impairment under diuretics. Whether or not albumin infusion exerts beneficial effect in those patients with reduced DMSA uptake remains to be determined.

## Background

A reduction of the renal ability to excrete sodium and free water and a decrease in renal perfusion and GFR are the three main renal function abnormalities in Cirrhosis [1]. However, assessment of renal function by common creatinine-based methods potentially are inaccurate in patients with liver cirrhosis [2]. Cirrhotic patients have several underlying conditions that contribute to falsely low serum creatinine concentrations including decreased creatinine production secondary to decreased hepatic creatine synthesis, increased tubular creatinine secretion, and decreased skeletal muscle mass. Serum creatinine level, measured creatinine clearance, and calculated creatinine clearance may all significantly overestimate GFR; the degree of GFR overestimation was a median of 95% in recent published studies [2,3].

Tc-99m dimercaptosuccinic acid (DMSA) is a radiopharmaceutical agent, which is eliminated by a complex interaction of glomerular filtration, peritubular uptake and tubular secretion [4]. About 50% of the injected dose accumulates in the cortex within I hour of injection and remains in the kidneys for 24 hours. This compound is useful for estimating "functional renal mass" being closely related to GFR [5]. Recently, we have shown that renal scintigraphy by DMSA uptake very useful method in evaluating the renal function (GFR) in patients with cirrhosis [[6], hepatology 2001; A 650]; 72% of patients with compensated liver cirrhosis had functional renal failure by DMSA uptake as compared to 86% in decompensated cirrhosis and none in healthy controls [6]. Early detection of renal dysfunction with DMSA renoscintigraphy would be clinically beneficial because it has been shown that clinical renal failure is a significant risk factor predictive of worse postransplant renal function, longer postransplant hospitalization and increased mortality [7,8]

Furosemide, a loop diuretic, inhibits chloride and sodium reabsorption in the thick ascending limb of the loop of Henle. but has no effect on the distal nephron [9]. It is rapidly absorbed from the gut, is highly bound to plasma proteins and is actively secreted from the blood into the urine by the proximal tubular cells. Once in the luminal compartment, furosemide is carried out with the luminal fluid to the loop of Henley, where it inhibits the Na+2Cl-K+ co-transport system located in the luminal membrane [9]. Since between 30–50% of the filtered sodium is reabsorbed in the loop of Henle using this transport system, furosemide has a high natriuretic potency. At high dosage, it may increase sodium excretion up to 30% of the filtered sodium in normal subjects. Furosemide increases also the synthesis of prostaglandin E2 by the ascending limb cells, and this effect is also related to its natriuretic effects since prostaglandins inhibit sodium reabsorption in the loop of Henle and NSAID impairs the diuretic and natriuretic effect of furosemide [10]. The onset of the action of furosemide is very rapid with peak effect occurring within 1–2 hours; the diuretic effect ends in 3–4 hours after administration.

The pharmacodynamic alteration that occurs in response to furosemide injection in cirrhotic patients remains undefined. Per drug excreted; there is much less sodium excretion in cirrhotics responsive to furosemide as compared to healthy controls and the response is even further blunted in resistant patients [11]. Caregaro et al showed that patients with compensated Child-A cirrhosis have sub clinical hypovolaemia and impaired natriuresis after saline loading, confirming that renal sodium handling abnormalities might precede ascites formation [12]. Whether or not DMSA uptake under diuretics identifies those patients who could develop renal impairment under diuretics remains to be determined. The aim of the present clinical study was to assess the renal functional mass by DMSA uptake before and after the administration of low (40 mg) and high dose (120 mg) of intravenous furosemide in patients with compensated liver cirrhosis without ascites and to determine whether DMSA uptake may identify patients who could develop renal impairment under diuretics and to further discriminate those patients within the child-A cirrhosis-patients.

## Methods

The study population included 18 cirrhotic patients. The cause and diagnosis of cirrhosis was identified based on clinical, biochemical and histological findings. Physical examination, nutritional status assessment including serum albumin, lymphocytes counts, body mass index (BMI), ascites (US) and the Child Push's classification were determined [13]. Patients were then divided randomly (arbitrary) into two groups. The first group (N = 8) includes patients with compensated cirrhosis and were given low dose intravenous 40 mg furosemide and the second group (N = 10) included patients with compensated cirrhosis and were given high dose 120 mg furosemide. Patients with systemic arterial hypertension, cardiac and renal disease, Patients with encephalopathy, bacterial infection and gastrointestinal hemorrhage in the two weeks before the study or those who had received nephrotoxic or non-steroidal anti-inflammatory drugs in the month before the study were excluded. Cirrhotic patients with ascites and severe renal failure (creatinin > 3 mg/dl) were not enrolled in the study due to short-term survival. None of the patients with organic renal failure was enrolled: the urinary sediment was normal, the proteinuria was lower than 500 mg/d and the size of the kidneys was normal as measured by ultrasound. Patients were admitted to the hospital, kept under low sodium diet (80 mEq/d) and all diuretics were withdrawn for 3 days. 24 hours Urine sample were taken to measure, volume, solute excretion, creatinine, protein, sodium and osmolarity. Fractional excretion of sodium was measured accordingly. Blood pressure was measured immediately after the injection, 12 and 24 hours later. Blood sample were taken to measure standard liver and renal biochemical function parameters. 2–3 mCi (100 MBq) of Tc 99 DMSA was given intravenously at base line (0 minute) and 90 minutes after the Furosemide administration (peak action of furosemide). Imaging was performed three hours later with posterior, anterior, oblique and lateral views. (Normal >15–20% uptake for each kidney = functional mass, Normal Global renal DMSA uptake >40%). Urine was collected for 8 hours after furosemide injection, with measurement of urinary sodium and volume. Creatinine clearance (cc/min) was calculated by the Cockroft-Gailt equation: (140-age (years) × patient weight (kg)/72 × Serum Cr (mg/dl) × 0.85 (If female) and by the 24-hours measured creatinine clearance method (UcrxU volume/Pcrx1440) [14]. The study was approved by an institutional ethics committee (Sieff Hospital Helsinki Committee) and each patient signed an informed consent.

## Statistics

Results were expressed, as mean ± SD. Demographic, clinical, and biochemical findings are categorized as continuous or categorical variables. Comparisons between variables before and after furosemide injection were done by χ-square test for categorical variables and by Wilcoxon signed rank test for continuous variables. Correlation coefficients were done by multiple regression analysis. Comparisons between groups for non-parametric data were done by Kruskal-Wallis analysis of variance. P value less than 0.05 was considered significant.

## Results

Eight patients with low dose furosemide (40 mg, group 1) and ten patients with high dose furosemide (120 mg, group 2) constitute the patients population of this study. 10 healthy peoples matched for age and sex served as controls. The diagnosis of liver cirrhosis was confirmed by liver biopsy in all cases. There were 10 men and 8 women (mean age 54 ± 8, range 40–71 years). Cirrhosis was related to chronic alcoholism in 7 patients, 4 patients had cirrhosis related to hepatitis C and B virus infection, 4 patients had primary biliary cirrhosis and 3 had cryptogenic cirrhosis. All patients in both groups had compensated cirrhosis with good synthetic function but without ascites. The intravenous injection of 120 mg of furosemide was not associated with any adverse effect such as hypotension or deterioration of renal function.

**Figure 1:**
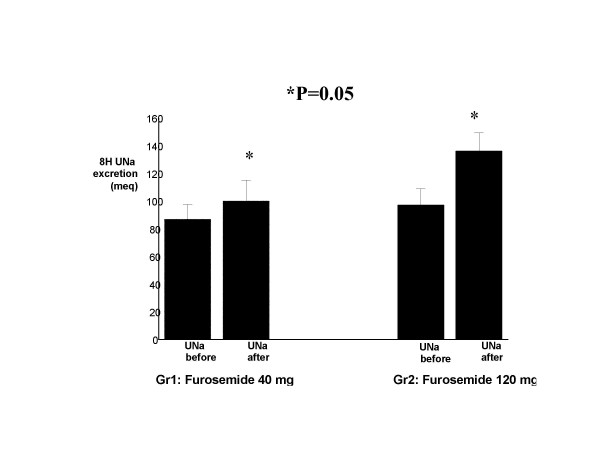
8-hour urinary sodium excretion in compensated cirrhotic patients before And after low and high dose furosemide injection (low dose: before (mean 87 ± 26, median 80, range 60–122); after: (mean 100 ± 36.6, median 96, range 42–147). High dose: before (mean 97 ± 32, median 107, range 37–128);after: (136 ± 37, median 127, range 92–212)

**Figure 2:**
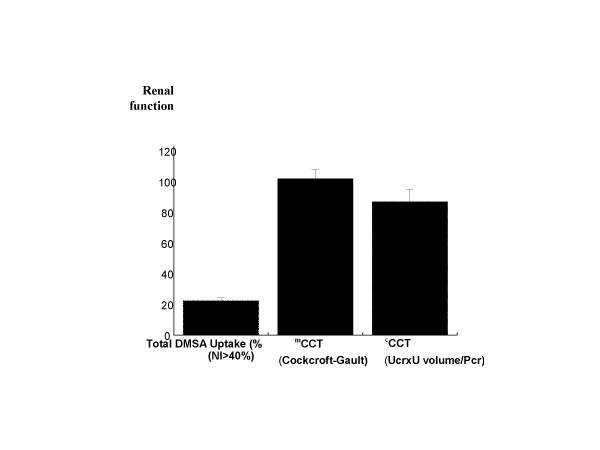
Renal functional mass in 18 compensated cirrhotic patients as assessed by global DMSA uptake (mean 22.5 ± 7.5%, median 22.5%, range 7%–33%), Calculated creatinine clearance (CCT, mean 87 ± 30, median 3, range 41–150), and Measured creatinine clearance (CCT, mean 101 ± 26, median 96, range 61–150)

As shown in Table 1, there was no significant difference between Gr 1 and Gr 2 for age, sex ratio, etiology of liver disease, MELD score and Child-Pugh's score. Serum creatinine, creatinine clearance, serum albumin, and international normalized ratio were also similar. We confirm in this study the correlation at baseline between DMSA and GFR that we observed previously in patients with cirrhosis and clinically normal renal function (hepatology 2001; A 650). Mean global DMSA uptake was 27.3 ± 4.4% in the healthy controls and 22.5 ± 7.5% in patients with compensated cirrhosis (P < 0.01). The Intravenous injection of Furosemide increased the mean renal DMSA uptake in 55% of patients with compensated cirrhosis and these changes persist up to 3 hours after injection. This increase was at the same extent in both groups (from 12.8% ± 3.8 to 15.2% ± 2.2 after 40 mg furosemide, +19% change in Gr 1) p < 0.001) and from 10.6% ± 4.6 to 13.5% ± 3.6 after120 mg furosemide, +27% change in Gr 2), p < 0.001), (Table 1) respectively. 5 patients (60%) in the first group improved the GFR by mean of 3.6% (delta increase, 12.3 ± 3.3 VS 15.9 ± 3.9, p < 0.003) and 3 patients had no changes whereas 5 patients in the second group did improve the GFR by 5.6% (delta increase, 7.7 ± 2.5 VS 13.3 ± 3.1 p < 0.001) and 5 patients with no changes. There seems to be a tendency for an increase in DMSA in the furosemide 120 mg group as compared to the 40 mg group. This is probably a type 2 error due to the small size of the population. None of the patients deteriorate the renal perfusion after furosemide injection. The 8-hours urinary sodium excretion after intravenous administration of furosemide increased significantly in 64% of patients and was higher in patients with 120 mg furosemide as compared with patients with 40 mg furosemide (136 ± 37 meq/l) VS 100 ± 36.6 meq/l, P = 0.05 (Fig-1), All patients secreted more than 80 meq/l. 80% of patients had a decreased GFR by DMSA (mean 22.5 ± 7.5% Vs 27.3 ± 4.4% in the healthy controls) in spite of normal GFR by calculated formula (101 ± 26 cc/min) and normal GFR by measured creatinine clearance (87 ± 30 cc/min, p < 0.001, Fig-2). There was a better correlation with calculated CCT but the method was significantly less accurate than DMSA uptake. Finally, a week correlation was observed between 8-hours urinary sodium changes and total basal DMSA uptake changes in both kidneys (r = +3.5, p < 0.5). Patients who did not increase DMSA uptake had a higher urinary sodium than patients who increased DMSA uptake but without statistical significance (mean 49 ± 53, median 38, range 5–148 Vs mean 25 ± 30, median 26, range 30–70, p < 0.1). The follow up visit one week later, showed the same creatinine level in both groups (serum creatinine 0.9 ± 0.15 in gr 1 VS 0.93 ± 0.19 in gr 2).

## Discussion

The present clinical study shows that intravenous injection of furosemide increased the renal functional mass by DMSA uptake in 55% of patients with compensated cirrhosis and these changes persist up to three hours after the injection of furosemide. In the remaining 45% of patients, DMSA uptake remains unchanged confirming the importance of using DMSA under diuretics to identify those patients who could develop renal impairment under diuretics and to further discriminate those patients within the child A cirrhosis-patients. Moreover, the study showed that intravenous furosemide increases the 8 hours urinary sodium excretion to levels above 80 meq/litre in 64% of patients and confirms previous finding that kidney function can be assessed accurately with quantitative SPECT of 99-Tc-DMSA uptake [6].

Daskalopoulos demonstrated that intravenous furosemide increased renal perfusion in 46% of cirrhotic patients with ascites and decrease renal perfusion and GFR in 54% [17]. However in that study renal perfusion was measured by P-aminohippurate clearance and was performed in deccompensated cirrhosis with ascites and not in compensated cirrhosis without ascites.

The mechanism by which furosemide improve the functional renal mass by DMSA uptake which reflect accurately the GFR remain unclear. Earlier observations pointed out that the renovascular effect of furosemide is partially dependent on the stimulation of the renal synthesis of vasodiladatory prostaglandin (prostacyclin = PGE2) induced by this diuretic and by counteracting the effects of vasopressor agents [15]. Another explanation is that furosemide at dose ranging from 40–120 mg did not inhibit the tubuloglomerular feedback mechanism which is involved in the maintenance of renal perfusion pressure during changes in arterial pressure in the post furosemide period [16]. It is now well recognized that furosemide pharmacokinetics are not altered by cirrhosis with normal serum albumin levels [18]. These patients with pre-ascitic cirrhosis have normal or reduced plasma renin and aldosterone levels [18].

The finding of an increase in the 8-hours urinary sodium above 80 meq/l in the majority of our patients with compensated cirrhosis following furosemide administration is similar to the finding by Sparhr et al [19] and can be explained by several factors: first there is increased delivery of furosemide to renal tubule by intravenous route, which is proportional to the increase in GFR. Second there is increase in the amount of sodium and water reaching the ascending of Henley's loops, the site of action of furosemide. [20]. The diagnosis of clinical renal failure in patients with cirrhosis carries a poor prognosis with a mortality rate of 40% [21]. Moreover, diuretic use in cirrhotic patients without ascites is associated with renal dysfunction. Therefore it is important to identify these patients reliably and rapidly, as liver transplantation may be needed early in these patients. The Clinical implication of this study is that it shows for the first time the safety of administering a single bolus of intravenous furosemide for patients with compensated cirrhosis without ascites and identify a subgroup of patients who could develop renal problems under diuretics and to further discriminate those patients within the child A-cirrhosis patients. This can help in the decision making for listing a patient early for liver transplantation. A second important issue is that DMSA uptake was more accurate parameter to quantify GFR than measured or calculated creatinine clearance that generally over-estimates the GFR in these patients. [[6], Fig 2]. Finally, the measurement of urine sodium after administration of intravenous furosemide may help in differentiating patients with lower treatment response to diuretics.

The reasons why 45% of our patients (3 patients in group 1 and 5 patients in group 2) did not increase DMSA uptake after furosemide injection remains unclear. Pinzani et al showed that transient decrease in renal function parameters (inulin) might be observed during 4 hours after drug injection, but without clinical consequences [20]. An alternate explanation may be that diuretics may increase systemic plasma renin activity over several hours independent of fluid loss and the increase in angiotensin II may impair renal blood flow by severe vasoconstriction. [22]. another explanation is impaired diuretic entree into the lumen, perhaps due to competition from other organic anion such as bile slats or due to low albumin levels [20]. However, the normal serum albumin in our patients implicate a normal or decreased volume of distribution and sufficient concentration of furosemide reaching the secretory site in proximal nephrons with concomitantly enough drug secreted into the urine. It should be noted also that the natriuretic response might reach a plateau at higher rate of diuretic excretion, presumably due to complete inhibition of the diuretic-sensitive carrier or channel [23]. Finally, the reason that only 55% of patients had an increased renal functional mass may be related to down regulation of cyclooxygenase-1 (COX-1)-derived prostaglandins in the kidneys of other patients [24]. Albumin infusion has been shown to improve the response to diuretics in patients with cirrhosis and ascites [25,26]. Whether the infusion of albumin in those patients with reduced DMSA uptake under diuretics prevent renal impairment or not remains to be determined.

The dose of intravenous furosemide was chosen according to the results obtained in a previous study evaluating furosemide kinetics and dynamics in patients with cirrhosis [11]. Urinary collection was performed during 8 hours because all the natriuretic effects of the drug occur within this time interval and because it has been shown that 8 hours urinary sodium excretion correlate strongly with 24 hours urinary sodium excretion [19]

Unfortunately we did not measure the plasma renin and plasma aldosterone levels before and after furosemide administration neither the urinary U-6-keto-PGF1a that reflects renal PGE2 synthesis. A future study incorporating these parameters as well as assessment of portal hypertension is warranted.

## Conclusion

We confirm previous findings that patients with cirrhosis and clinically normal renal function can have moderate to severe impaired GFR (as assessed by DMSA) despite the presence of normal creatinine. A single furosemide injection increases renal functional mass as reflected by DMSA in 55% of patients with compensated cirrhosis and reduced DMSA uptake in 45% of patients. This confirms the eventual importance of using DMSA uptake under diuretics to identify those patients who could develop renal impairment under diuretics. Whether or not albumin infusion exert beneficial effect in those with reduced DMSA uptake remains to be determined.

## Competing interests

We disclose any financial competing interests but also any non-financial competing interests that may cause them embarrassment were they to become public after the publication of the manuscript.

## Authors' contributions

All authors read and approved the final manuscript

NA Wrote the manuscript and design, analyze, and interpreted the data.

MK Carried out the DMSA uptake and patients selections

YM Participate in its design and coordination

MG Carried out the DMSA uptake

OH participate in drafting the manuscript and in its design

SS Conceived of the study, and participated in its design and coordination

## Pre-publication history

The pre-publication history for this paper can be accessed here:



## Figures and Tables

**Table 1:** patient characteristics:

Characteristics	Group 1 Furosemide 40 mg (n = 8)	Group 2 Furosemide1 120 mg (n = 10)	P value
Age (years)	56.5 ± 9.6	54 ± 9.0	NS
Sex ratio (M/F)	5/3	6/4	NS
Etiology : Alcoholic	3	4	NS
Non alcoholic	5	6	NS
Serum albumin(g/L)	39 ± 4	40 ± 5	NS
INR	1.09 ± 0.1	1.1 ± 0.1	NS
Bilirubin (micromol/L)	20.5 ± 34.9	18.8 ± 8.5	NS
Child-Pugh's score	5–6	5–6	NS
MELD score	7	8	NS
Serum sodium(mEq/L)	136 ± 1.0	137 ± 1.6	NS
Median (range)	136 (134–138)	136 (133–139)	
Serum creatinine (micromol/L)			
Base line	77.8 ± 14	81.3 ± 15	NS
After 1 week follow up	79.6 ± 13	82.2 ± 16.8	NS
Creatinine clearance (ml/min)	82.6 ± 30.4	89.8 ± 32.0	NS
Median (range)	73 (56–142)	92 (41–150)	
8-hours UNa (before, median, range)	87 ± 26 (80, 60–122)	97 ± 32 (107, 37–128)	NS
8-hours UNa (After mEq, median, range)	100 ± 36 (96,42–147)	136 ± 37 (127, 92–213)	0.05
% Change	+15%	+40%	0.01
DMSA Uptake (Nl > 20%)			
Before Furosemide (median, range)	12.8 ± 3.8 (14, 3–17)	10.6 ± 4.6 (10, 3–21)	0.001
After Furosemide (median, range)	15.2 ± 2.2 (16, 10–20)	13.5 ± 3.6 (15, 6–21)	0.001
% Change	+19%	+27%	
	p < 0.001	p = <0.003	
